# Pharmacokinetics and modeling of immune cell trafficking: quantifying differential influences of target tissues versus lymphocytes in SJL and lipopolysaccharide-treated mice

**DOI:** 10.1186/1742-2094-9-231

**Published:** 2012-10-03

**Authors:** William A Banks, Michael L Niehoff, Nicholas M Ponzio, Michelle A Erickson, Steven S Zalcman

**Affiliations:** 1 GRECC, Veterans Affairs Puget Sound Health Care System, Seattle, WA, USA; 2 Division Gerontology and Geriatric Medicine, Department of Medicine, University of Washington School of Medicine, Seattle, USA; 3 VAPSHCS, Rm 810A, 1660 S. Columbian Way, Seattle, WA, 98108, USA; 4 Division of Geriatric Medicine, Department of Internal Medicine, Saint Louis University School of Medicine, Saint Louis, MO, USA; 5 Department of Pathology and Laboratory Medicine, UMDNJ-New Jersey Medical School, Newark, USA; 6 Department of Pharmacological and Physiological Sciences, Saint Louis University School of Medicine, Saint Louis, USA; 7 Department of Psychiatry-UMDNJ-New Jersey Medical School, Newark, USA

**Keywords:** Blood–brain barrier, Brain, Cellular trafficking, Encephalitis, Immune, Lymphocytes, Multiple sclerosis, Neuroinflammation, Neuroimmune, Pharmacokinetics

## Abstract

**Background:**

Immune cell trafficking into the CNS and other tissues plays important roles in health and disease. Rapid quantitative methods are not available that could be used to study many of the dynamic aspects of immune cell-tissue interactions.

**Methods:**

We used pharmacokinetics and modeling to quantify and characterize the trafficking of radioactively labeled lymphocytes into brain and peripheral tissues. We used variance from two-way ANOVAs with 2 × 2 experimental designs to model the relative influences of lymphocytes and target tissues in trafficking.

**Results:**

We found that in male CD-1 mice, about 1 in 5,000 intravenously injected lymphocytes entered each gram of brain. Uptake by brain was 2 to 3 times higher in naïve SJL females, but uptake by spleen and clearance from blood was lower, demonstrating a dichotomy in immune cell distribution. Treatment of CD-1 mice with lipopolysaccharide (LPS) increased immune cell uptake into brain but decreased uptake by spleen and axillary nodes.

**Conclusions:**

Differences in brain uptake and in uptake by spleen between SJL and CD-1 mice were primarily determined by lymphocytes, whereas differences in uptake with LPS were primarily determined by lymphocytes for the brain but by the tissues for the spleen and the axillary lymph node. These results show that immune cells normally enter the CNS and that tissues and immune cells interact in ways that can be quantified by pharmacokinetic models.

## Background

The central nervous system (CNS) is an immune-privileged tissue. Its invasion by immune cells during autoimmune diseases, such as multiple sclerosis, underlies much of the pathology of those diseases and is key to their treatment [1]. Immune cell invasion of the CNS is increasingly viewed as important to the development and progression of a wide range of diseases including neuroAIDS [2,3], trauma to the CNS [4], stroke [5], epilepsy [6], and neurodegenerative diseases [7]. More recently, the CNS has emerged as an immune-active tissue under normal physiologic conditions, rather than merely in disease states [8,9]. Similarly, it is now widely accepted that immune cells routinely migrate into the CNS in small but undetermined amounts at a low but undetermined rate [10]. The possibility that immune cell trafficking into the CNS may occur normally is suggested by the finding that immune cells readily cross brain endothelial cells when studied with *in vitro* models of the blood–brain barrier (BBB) [11]. The development of progressive multifocal leukoencephalopathy in patients treated with antibodies that prevent or retard immune cell entry into the CNS has suggested that the CNS may normally be under immune surveillance from cells trafficking into the CNS [12].

The degree to which immune cells normally cross the BBB is difficult to address with the methods classically employed. Almost all *in vivo* studies of immune cell trafficking into the CNS have used models that activate the immune system in imitation of a disease state, as exemplified by induction of experimental allergic encephalitis in the study of multiple sclerosis [13,14], or invoke neuroimmune responses with cytokines, lipopolysaccharide (LPS), or viruses [2,3,15,16]. Classic studies have demonstrated that immune cell entry into the CNS involves an intimate cross talk between the brain endothelial cells comprising the BBB and the circulating immune cells. The difficulty in quantifying immune cell uptake, however, makes it difficult to determine the temporal course of uptake, the relative distribution of immune cells among tissues, and whether it is the immune cell or the target tissue that initiates or dominates trafficking. Here, we adapt pharmacokinetic techniques and modeling to quantify lymphocyte uptake and distribution patterns. With these methods, we were able to quantify the rate at which lymphocytes were taken up by brain and peripheral tissues, clearance rates from the circulation, the effect of genetic strain and activation of the innate immune system on lymphocyte distribution, and the relative contributions made by lymphocytes and target tissues in that distribution.

## Methods

All animals were studied according to approved protocols that conformed to the NIH Guide for the Care and Use of Laboratory Animals and approved by the local animal use committee (John Cochran VA, St. Louis, MO. USA). Two strains of outbred mice, male CD-1 from our in-house colony (VA, St. Louis) and female SJL/J mice (Jackson Laboratories, Bar Harbor, ME), 8 to 12 weeks of age, were used to harvest lymphocytes and perform the *in vivo* injection studies.

### Lymphocyte harvest

Cervical-node mixed lymphocytes were harvested from two or three SJL/J or CD-1 mice, which were anesthetized with an intraperitoneal (i.p.) injection of 40% ethyl carbamate. Prior to harvesting the lymph nodes, the vascular space of the head and neck was washed free of blood. Each mouse was placed in a supine position and the skin removed from the neck to expose the right and left jugular veins. The thorax was opened by cutting through the sternum from the epigastric region of the abdomen to the sternal notch. Both jugular veins were severed. The descending thoracic aorta was clamped with a hemostat. The head and neck were then perfused with 20 ml of lactated Ringer’s solution (LR) injected into the left ventricle of the heart over a one minute interval, using a 20 ml syringe with a 20 g needle.

Four cervical lymph nodes were then dissected clean and harvested from each mouse and placed in a petri dish with normal saline (NS) at room temperature. Forceps were used to tease apart the nodal tissue to release lymphocytes into the NS solution, and the cell suspension placed in a 12 × 75 mm polypropylene tube. The cells were centrifuged at 300 rpm for 3 min to remove tissue fragments. The supernatant containing the lymphocytes was transferred to a clean tube and centrifuged at 1,000 rpm for 10 min to pellet the lymphocytes. The supernatant was discarded and the cells washed twice with 1 ml 0.25 M phosphate buffer at pH 7.5 (PB) to remove the NS. The cells were then resuspended in 200 μl PB and radioactively labeled as described later.

### Lymphocyte iodination

To iodinate the harvested lymphocytes, two IodoBeads (Pierce, Inc., Rockford, IL) were washed twice with 500 μl PB for 30 s each wash, and placed in a 12 × 75 mm glass tube with 200 μl PB. A total of 2 mCi ^131^I (Perkin Elmer, Shelton, CT) was added and incubated at room temperature for 5 min. The cells were resuspended by lightly vortexing, 200 μl of the suspension was added to the iodination material, and the mixture was incubated at room temperature for 20 min. The iodination mixture was vortexed to resuspend the cells, and all liquid was removed from the reaction tube to a clean 12 × 75 mm glass tube.

To remove the free iodine, the iodination material was centrifuged at 1,000 rpm for 10 min to pellet ^131^I-lymphocytes (I-lymphocytes). The supernatant was discarded and the ^131^I-lymphocytes washed at least three times with 500 μl of lactated Ringer’s solution containing 1% bovine serum albumin (LR-BSA). The ^131^I-lymphocytes were resuspended in 200 μl of LR-BSA and counted on a standard hemocytometer. To determine the percentage of ^131^I incorporated, a 15 μl aliquot of the ^131^I-lymphocyte suspension was centrifuged at 20,000*g* for 20 min, and the amount of radioactivity in the pellet and supernatant counted in a gamma counter (Wallac, Inc., Gaithersburg, MD). The ^131^I-lymphocytes typically required three washings with LR-BSA to remove free ^131^I and had an incorporation ≥90%. This iodination procedure resulted in a mean specific activity of 53.3 ± 10.97 cpm/lymphocyte (range 19.4 to 94 cpm/lymphocyte) and a mean cell count of 8.9 × 10^4^ ± 2.1 × 10^4^ cells/0.1 ml (range 2.5 × 10^4^ to 18 × 10^4^ cells/0.1 ml) with the total level of radioactivity being 42,177 ± 10,914 cpm/μl (range 12,400 to 87,240 cpm/μl). There were no apparent differences in the iodination results between the CD-1 and SJL/J lymphocytes.

### *In vivo*^131^I-lymphocyte injection studies

These studies were used to determine the clearance and distribution of ^131^I-lymphocytes after their intravenous (i.v.) injection. Mice (*n* = 7/time point) were anesthetized with 40% ethyl carbamate and the skin excised from the neck between the sternal notch and the chin. The cervical fat pad was pulled back to expose the left jugular vein for i.v. injection. The ^131^I-lymphocytes were injected into the left jugular vein in a volume of 0.2 ml of LR-BSA at a concentration of 150,000 cpm/mouse. The ^131^I-lymphocyte solution was mixed vigorously prior to filling the syringe to resuspend the cells. An injection check of 0.2 ml, representing the total cpm injected into each mouse, was also placed into a 12 × 75 mm tube and the level of radioactivity determined. At post-injection study times of 5, 15, 30, 90, or 150 min, a midline laparotomy was performed to expose the abdominal aorta. The aorta was severed and blood collected in a preweighed 0.6 ml microcentrifuge tube. The tube was weighed again to determine the amount of blood collected and the blood was allowed to clot at room temperature, and then centrifuged at 20,000*g* for 20 min. The serum was then removed and its volume determined and the remaining volume of the red blood cell (RBC) pellet calculated. The radioactivity representing the ^131^I-lymphocytes in the pellet was determined in the gamma counter. Results are expressed as cpm/μl of RBC pellet or as the percentage of the injected dose per ml of pellet (%Inj/ml).

Immediately following the blood collection, a thoracotomy was performed with a midline incision through the sternum to the sternal notch. Both jugular veins were severed, and the descending thoracic aorta clamped. Cardiac perfusion with 20 ml of LR was accomplished as described, to wash out all blood components from the vascular space of the head and neck. The cervical nodes, axillary nodes, and spleen were collected and weighed. The mouse was then decapitated, the parietal skull removed, and the brain exclusive of the pineal body and pituitary, removed and weighed. The brain was placed in 12 × 75 mm polytubes with 1 ml of LR-BSA. The brain and peripheral tissues were centrifuged at 4,800 rpm for 10 minutes to pellet the tissue into the bottom of the tube, then the level of radioactivity determined with a gamma counter.

Each brain was homogenized using a Polytron (Brinkmann Instruments, Westbury, NY) with a 7 mm probe operated at setting 20 for 15 s. The homogenate was placed in a microfuge tube and centrifuged at 20,000*g* for 20 minutes to pellet all cellular material. The supernatant and pellet were separated and the radioactivity in each determined with the gamma counter.

To verify that the ^131^I-lymphocytes were separated into the RBC and the brain pellets during centrifugation, processing controls (*n* = 2) were performed in which ^131^I-lymphocytes (150,000 cpm) were added to arterial blood or to whole brain that had the vascular contents washed out; these samples were from normal CD-1 mice. These were then processed as above and the level of radioactivity determined in the blood RBC pellet and serum and the brain pellet and supernatant. The RBC pellet contained 87% of the total level of radioactivity, while the brain pellet contained 89% of the total level of radioactivity.

Uptake by brain and other tissues were quantified by expressing results in two ways. For the brain or tissue/RBC pellet ratios, the percentage of injected dose taken up per g of tissue was calculated. The former is independent of clearance from blood and the latter is useful for calculating global effects on tissue uptake. For the brain/RBC ratios and tissue/RBC ratios, the levels of radioactivity in the brain pellet and the RBC pellet were used to calculate

(1) $$\text{Brain/RBC ratio}=\left[\left(\text{cpm/g of brain pellet}\right)/\text{(cpm/μl of RBC pellet})\right]$$

in units of μl/g. Similarly, the tissue/RBC ratios were calculated for spleen, cervical nodes, and axillary nodes. These were plotted against time to determine the temporal pattern of ^131^I-lymphocyte uptake by the brain or peripheral tissues.

For the percentage of injected dose taken up by the brain, the percentage of the i.v. injected dose of ^131^I-lymphocytes taken up by a gram of brain (%Inj/g) was calculated from the equation

(2) $$\text{\%Inj/g= }\left[\left(\text{tissue cpm/Inj}\right)\text{/tissue wt}.\right]*100$$

where tissue cpm is the level of radioactivity measured in the specific tissue, and tissue wt. is the weight in grams of the specific tissue.

### Clearance from blood

The percentage of the intravenously injected dose of ^131^I-lymphocytes per ml of arterial blood (%Inj/ml) was calculated from the equation:

(3) $$\text{\%Inj/ml= }100\left(\text{Cp/Inj}\right)$$

where Cp is the cpm/ml of the RBC pellet and Inj is the total cpm injected i.v. The log %Inj/ml was plotted against time and the inverse of the slope of the linear portion multiplied by 0.301 to obtain the half-time clearance. The inverse of the antilog of the intercept was multiplied by 100 to give the initial volume of distribution (Vd).

To examine regional brain uptake, this study compared the relative permeability of various brain regions to ^131^I-lymphocytes. Blood and brains were collected 30 min after injection. The brain was dissected into 11 brain regions (olfactory bulb, frontal cortex, parietal cortex, occipital cortex, striatum, hippocampus, thalamus, hypothalamus, cerebellum, midbrain, pons-medulla) after the method of Glowinski and Iversen [17]. Regions were weighed and their levels of radioactivity determined. Values for whole brain were calculated based on the summations of the regional brain weights and the regional radioactivity levels of the brain regions.

### Autoradiography and immunohistochemistry of tissues

These studies were performed to confirm that radioactivity taken up by brain was associated with lymphocytes. The ^131^I-lymphocytes were injected into the left jugular vein in a volume of 0.2 ml of LR-BSA at a concentration of 34,000 cpm/0.2 ml and 18 × 10^4^ cells/0.2 ml. Five minutes post-injection, cardiac washout was performed as described. Four cervical nodes were removed, placed in 1 ml of PB, and then teased apart with forceps. The cellular suspension was centrifuged at 300 rpm for 10 min to remove tissue particles. The lymphocyte-containing supernatant was then centrifuged at 1,000*g* for 15 minutes to pellet the ^131^I-lymphocytes. The pellet was resuspended in 100 μl fresh LR-BSA, then slide smears prepared using 15 μl of I-lymphocyte suspension on each slide. The slides were dried overnight, heat fixed at 60°C for 20 min then stored at 4°C until stained.

In a companion study to visualize ^131^I-lymphocytes in the brain parenchyma, CD-1 or SJL mice were anesthetized and prepared for i.v. injection, as before. ^131^I-lymphocytes were i.v. injected at a concentration of about 4.5 × 10^6^ CPM/0.2 ml and 2,000 × 10^4^ cells/0.2 ml, with CD-1 mice receiving ^131^I-lymphocytes from CD-1 mice and SJL mice receiving ^131^I-lymphocytes from SJL mice. Thirty minutes after i.v. injection, the thorax was opened and cardiac washout was performed to clear the brain vasculature of blood. The mice were decapitated, the brain removed and the central region isolated by making two coronal cuts, one anterior to the hypothalamus, and a second cut posterior to the occipital cortex. This central brain region was then flash frozen in isopentane (Sigma Chemical, St. Louis, MO) and dry ice. The frozen specimen was mounted on a cryostat chuck at −16°C using OTC compound. Following a 2 hour equilibration period, 20 μm sections were cut using a Reichert-Jung Cryocut 1800 microtome (Leica Microsystems Inc., Bannockburn, IL). Every four to ten sections were mounted on glass slides, and dried at room temperature to adhere sections to the slides; the slides were stored at −70°C until stained. Slides of the cervical-node lymphocyte smear or brain sections were brought to room temperature and allowed to dry. The slides were fixed in acetone: alcohol, 75:25, for 5 min, then washed in phosphate buffered saline (PBS). The sections were then blocked in 5% BSA in PBS for 30 minutes and washed in PBS; then endogenous peroxidase was blocked for 5 min using PBS with 0.3% H_2_O_2_ and 0.3% BSA. After washing in PBS, the sections were incubated with primary purified rat anti-mouse CD45 (leukocyte common antigen, Ly-5) antibody, CD45, 1:50 dilution (BD Pharmingen, San Jose, CA) in PBS overnight at 4°C. Slides were then washed with PBS and incubated for 30 min at room temperature with 1:200 dilution of biotinylated anti-rat IgG (Vector Laboratories, Burlingame, CA). Slides were washed with PBS and incubated with Vectastain ABC reagent (Vector Laboratories, Burlingame, CA) for 30 min. Slides were washed in PBS and stained with DAB solution (Vector Laboratories, Burlingame, CA) with nickel solution for 8 min. The reaction was stopped with PBS, and the slides rinsed well with water, then counter-stained with hematoxylin (Sigma Chemical, St. Louis, MO) for 30 s, rinsed with water and allowed to dry at room temperature.

Slides for brain and lymph nodes were then processed for autoradiography by dipping each slide in a 50% solution of Kodak Emulsion, Type NTB (Kodak, Rochester, NY) at 45°C for 10 s, then allowed to dry at room temperature in the dark. The slides were then sealed in light-tight boxes and stored at 4°C for 3 days. Following a 3 day exposure time, slides were processed using Kodak D-19 developer and fixer, dehydrated with graded alcohols and xylene, and coverslipped. Autoradiographic slides were examined using a Nikon Eclipse 80i microscope (Nikon Instruments Inc., Melville, NY), and photomicrographs were taken using a Photometrics Cool Snap cf camera (Roper Scientific Photometrics, Tucson, AZ) and MetaMorph v 6.1 software (Universal Imaging Corp., Downingtown, PA).

### Capillary depletion

This method was used to distinguish full penetration of the capillary wall with entry into the brain parenchymal space from association with the luminal surface of the capillary. In male CD-1 mice (*n* = 2), the relative distribution of ^131^I-lymphocytes between the cerebral cortex parenchyma and the capillaries was determined by the method of Triguero *et al*. [18] as modified for mice by Gutierrez *et al*. [19]. Following anesthesia, the left jugular vein was exposed as described above. Using a 1 ml syringe, 0.2 ml of LR-BSA containing 1 × 10^6^ cpm of ^131^I-lymphocytes was injected into the jugular vein. Thirty minutes later, the abdomen was opened and blood collected from the abdominal aorta. The RBC pellet was collected as described previously and the level of radioactivity determined. The thorax was opened, the thoracic descending aorta clamped, the left and right jugular veins severed, and the brain flushed of its intravascular contents by injecting 20 ml of LR solution over 1 minute into the left ventricle of the heart. The mouse was decapitated and the brain harvested as described above. The cerebral cortex was isolated, weighed, and placed in ice-cold physiological buffer (10 mM HEPES, 141 mM NaCl_2_, 4 mM KCL, 2.8 mM CaCl_2_, 1 mM MgSO_4_, 1 mM NaH_2_ PO_4_, and 10 mM D-glucose, adjusted to pH 7.4). The cortex was then homogenized in 0.8 ml of physiological buffer using a glass tissue grinder (ten vertical strokes). A dextran solution, 1.6 ml of a 26% solution in the physiological buffer, was added to the homogenate, mixed vigorously, and homogenized with three additional vertical strokes. The homogenate was centrifuged at 5,400*g* for 15 minutes at 4°C in a swing bucket rotor. The pellet containing the brain vasculature and the supernatant consisting of the brain parenchyma were carefully separated and the radioactivity of each component determined with a gamma counter. The parenchyma/pellet and capillary/pellet ratios (μl/g) were calculated by the equation:

(4) $$\text{Fr/pellet= }\left(\text{cpm Fr}\right)/\left(\text{wt}.\right)\left(\text{cpm/μl pellet}\right)$$

where cpm Fr is the cpm in either the parenchyma or supernatant fraction, wt. is the weight of the cortex, and cpm/μl pellet is the level of cpm in a microliter of RBC pellet.

To confirm that capillary depletion was an appropriate method for lymphocytes, processing controls for capillary depletion (*n* = 2) were performed by adding 1.5 × 10^5^ CPM ^131^I-lymphocytes to a tube containing arterial blood. This blood was then added to a brain in which the vascular contents had been removed by washout with LR, as described previously. The blood and brain were then processed, as described previously, for capillary depletion and the level of radioactivity in the pellets and supernatants determined in the gamma counter. The ratios (Fr/pellet) for parenchyma (0.57 ± 0.14) and capillary (0.82 ± 0.22) were not statistically different from each other (*n* = 3/group, *P* = 0.3758) with about 41% of the lymphocytes segregating with the parenchyma fraction.

### Lymphocyte uptake by CD-1 and SJL/J mice

This study compared these two strains for ^131^I-lymphocyte clearance from blood, brain distribution, and uptake by peripheral tissues. Lymphocytes from either CD-1 or SJL/J mice, *n* = 2 or 3 per strain, were harvested and iodinated as described, and i.v. injection studies performed in both CD-1 and SJL/J mice. A 2 × 2 design was employed, where iodinated lymphocytes from CD-1 mice were injected into CD-1 or SJL/J mice, *n* = 7/strain, and iodinated lymphocytes from SJL/J mice were injected into CD-1 or SJL/J mice, *n* = 7/strain. Blood from the abdominal aorta was collected 30 minutes after the injection, and washout performed as described previously. The cervical and axillary nodes, the spleen, and the brain were collected. The number and weight of each type of lymph node was recorded, and the weight per node calculated. The spleen and whole brain were weighed. Blood and tissues were processed as described previously and the level of radioactivity in each measured with a gamma counter. The tissue/RBC ratios and clearance from blood (%Inj/ml) were determined as described previously.

The effect of activation of the innate immune system with LPS on ^131^I-lymphocyte clearance, uptake, and distribution in male CD-1 mice was studied. CD-1 mice (*n* = 3) were treated with either i.p. injections of LPS or 0.25 M phosphate buffer, pH 7.5 (PB). The LPS (Sigma Chemical Co., St. Louis, MO) was prepared from *Salmonella typhimurium* and administered to each mouse in a dosage of 3 mg/kg at time 0, 6 h, and 24 h. Four hours after the 24 h i.p. LPS or PB injection, the mice were anesthetized, the vasculature of the head and neck washed free of blood, and lymphocytes harvested and iodinated as described previously.

Other CD-1 mice were treated with i.p. injections of either LPS or PB at time 0, 6 h and 24 h. Four hours after the 24 h i.p., LPS or PB injection, a 2 × 2 designed study was accomplished, where ^131^I-lymphocytes from LPS-treated mice were injected into LPS- (*n* = 7) or PB- (*n* = 6) treated mice, and ^131^I-lymphocytes from PB-treated mice were injected into LPS- (*n* = 5) or PB- (*n* = 7) treated mice. The mice were anesthetized, the jugular veins exposed, and the ^131^I-lymphocytes injected into the left jugular vein in a volume of 0.2 ml of LR-BSA at a concentration of 150,000 cpm/mouse. An injection check of 0.2 ml was placed into a 12 × 75 mm tube and the level of radioactivity determined. At the post-injection study time of 30 min, a midline laparotomy was performed, the abdominal aorta severed and blood collected in a 0.6 ml microcentrifuge tube. The vasculature of the head and neck was washed free of blood and the cervical nodes, axillary nodes, spleen, and whole brain were collected as previously described. The blood and tissues were processed as described previously and the level of radioactivity determined with a gamma counter. The tissue/RBC ratios and %Inj/ml were calculated as previously described.

### Statistical analysis

Statistical calculations were performed with Prism 5.0 software (GraphPad Software, San Diego, CA). Means are reported with their standard errors and the number of animals (*n*) per group. Two group comparisons were performed using Student’s two-tailed, unpaired *t* test with comparisons considered significant at the *P* < 0.05 level. For time curve comparisons, a two-way analysis of variance (ANOVA) was used to compare the independent variables followed by the Bonferroni post-test to compare values at individual times. For the 2 × 2 designs, a two-way ANOVA was used to compare the two independent variables (the source of the cells (Cells) and the mouse into which they were injected (Mouse)) followed by one-way ANOVA and Newman-Keuls post-test to compare the four values with each other.

## Results

**Figure 1:**
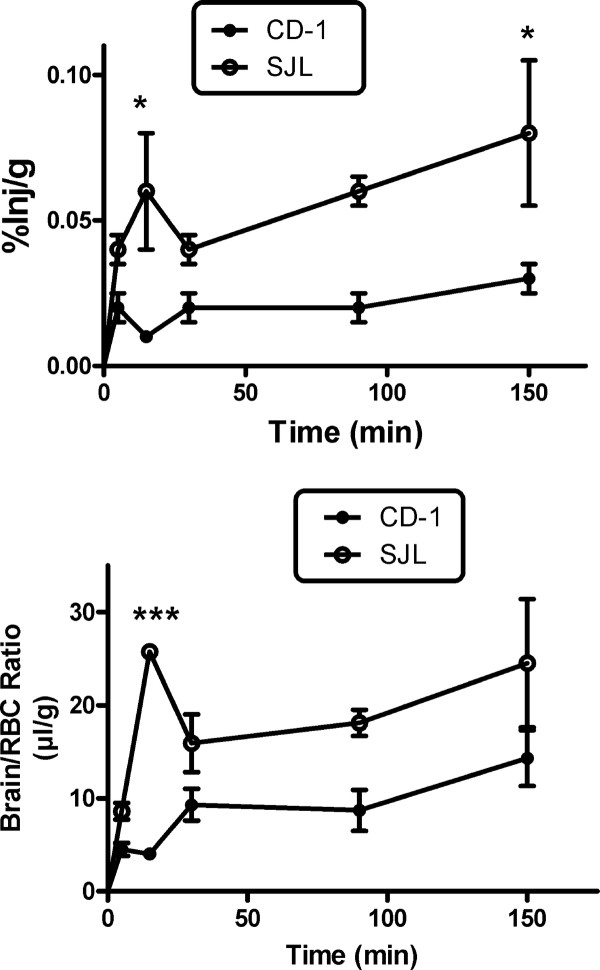
**Uptake over time of **^**131**^**I-lymphocytes into brain of CD-1 male and SJL female mice.** Upper panel expresses results as %Inj/g of brain tissue and lower panel shows results expressed as brain/RBC ratios. Uptake by SJL mice was 2 to 3 times greater than in CD-1 mice.

The uptake of ^131^I-lymphocytes by the brain in CD-1 and SJL mice is shown in Figure 1. These results show that ^131^I-lymphocytes were taken up by the brains of both CD-1 and SJL mice. The top panel compares results expressed as %Inj/g (*n* = 4/group). A two-way ANOVA showed an effect of mouse strain (*F*(1, 30) = 27.0, *P* < 0.0001), but not of time or interaction. The Bonferroni post-test compared CD-1 and SJL mouse strains at each of the various times and found statistical differences at 15 min (*P* < 0.05) and 150 min (*P* < 0.05). The lower panel compares the results expressed as brain/RBC pellet ratios. The two-way ANOVA showed an effect of strain (*F*(1,30) = 34.9, *P* < 0.0001), time (*F*(4,30) = 5.52, *P* < 0.005)), and interaction (*F*(4,30) = 2.96, *P* < 0.05). The Bonferroni post-test comparing CD-1 and SJL mice at each of the various times found a statistical difference at 15 min (*P* < 0.001). These results show that lymphocytes were taken up by the brain and rapidly achieved steady state, and that lymphocyte uptake by brain was greater for SJL mice than for CD-1 mice.

Capillary depletion was performed in male CD-1 mice to determine whether the brain retention of ^131^I-lymphocytes found in Figure 1 was because they crossed the BBB completely to enter the parenchyma of the brain or were merely retained by brain endothelial cells. Vascular washout was first performed 30 min after the i.v. injection of the ^131^I-lymphocytes, a procedure that removes cells and substances loosely bound to the luminal surface of the brain’s capillary bed [20]. After that, the brain was prepared as outlined previously to produce parenchymal and capillary fractions. The parenchyma/RBC ratio was determined to be 28.5 ± 1.4 μl/g, which was significantly higher than the capillary/RBC ratio of 6.7 ± 2.3, *n* = 2 μl/g, *P* = 0.0153. This shows that 81% (100(28.5)/(28.5 + 6.7) = 81) of the ^131^I-lymphocytes taken up by the brain had crossed the capillary wall of the BBB completely and had entered the brain parenchyma.

**Figure 2:**
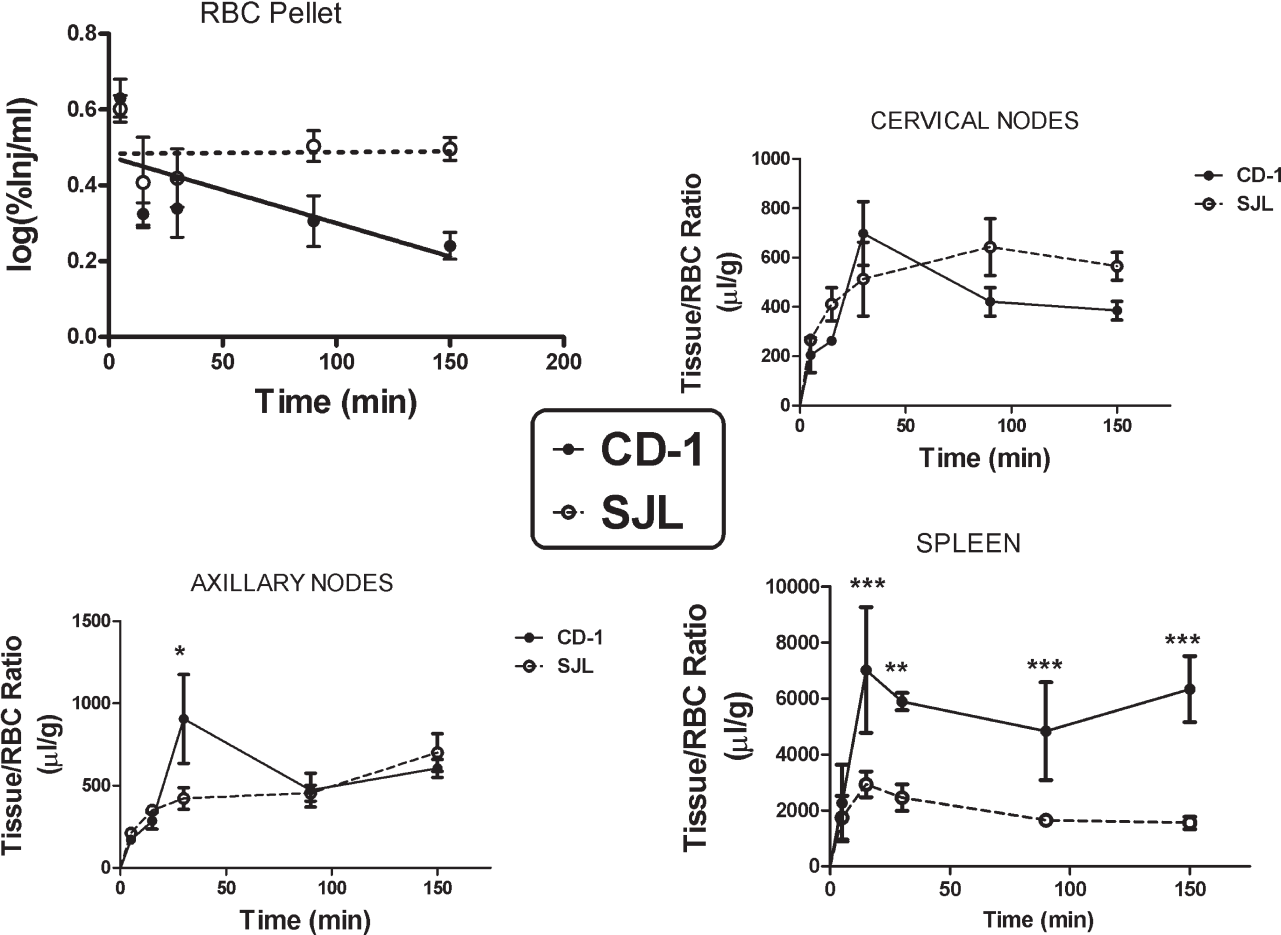
**Comparison between CD-1 males and SJL females of clearance from blood and uptake by immune tissues of **^**131**^**I-lymphocytes.** Upper left panel shows clearance of ^131^I-lymphocytes from the circulation. The half-time clearance for CD-1 mice was 171 min, but clearance was not measurable for SJL mice. Upper right panel shows that uptake of ^131^I-lymphocytes into cervical nodes was high in both SJL and CD-1 mice and did not differ between the strains. Lower left panel showed high uptake of ^131^I-lymphocytes by axillary nodes was similar between SJL and CD-1 mice except for the 30 min time point when CD-1 mice had a higher uptake. Lower right panel shows that spleen uptake was the highest of all the tissues examined and that uptake by CD-1 mice was about 2 to 3 times higher than by SJL mice.

Figure 2 (upper left panel) shows the log(%Inj/ml) of ^131^I-lymphocytes in CD-1 and SJL/J mice at 5 to 150 min after i.v. injection. In CD-1 mice, the ^131^I-lymphocytes were cleared from blood with time, as shown by a significant relation between log(%Inj/ml) and time, *r* = 0.577, *n* = 19, *P* = 0.0097. The half-time clearance rate from blood for these ^131^I-lymphocytes was calculated to be 171 min. In SJL/J mice, the level of ^131^I-lymphocytes in blood was constant, excepting for the first time point, suggesting that a steady state between entry and exit of the circulation was quickly established. The Vd for both strains was about 32 ml, demonstrating a large volume of distribution or significant tissue sequestration of the ^131^I-lymphocytes. Calculations of %Inj/g are dependent on %Inj/ml and so are influenced by differences in clearance of ^131^I-lymphocytes. Therefore, subsequent analyses comparing the strains were conducted using tissue/RBC ratios, which are less influenced by clearance.

Figure 2 compares CD-1 and SJL tissue/RBC ratios for cervical lymph nodes (upper right panel, no statistically significant differences), axillary nodes (lower left panel), and spleen (lower right panel). For cervical nodes, a two-way ANOVA showed an effect of time (*F*(4,30) = 6.40, *P* < 0.001), but not strain or interaction; the Bonferroni post-test found no differences between strains at any time. The two-way ANOVA for axillary nodes found an effect for time (*F*(4,30) = 7.90, *P* < 0.001)), but no effect for strain or interaction; the Bonferroni post-test found a difference between CD-1 and SJL at 30 min. The two-way ANOVA for spleen found differences for time ((*F*4,30) = 7.93, *P* < 0.001), strain (*F*(1,30) = 81.3, *P* < 0.0001), and interaction (*F*(4,30) = 4.17, *P* < 0.01); the Bonferroni post-test showed differences between SJL and CD-1 at 15 min (*P* < 0.001), 30 min (*P* < 0.001), 90 min (*P* < 0.01), and 150 min (*P* < 0.001). These results demonstrate that spleen, brain, and clearance from blood, but not cervical or axillary nodes, showed a difference between the two strains of mice. These results also show tissue-dependent differential effects of mouse strain in that CD-1 mice, by comparison with SJL mice, had less uptake into brain but more uptake into spleen.

Cervical node, axillary node, and spleen weights from CD-1 and SJL/J mice were compared. The wet weights of nodes of SJL/J mice were found to be significantly greater than those from CD-1 mice (cervical node: CD-1 = 5.2 ± 0.4 mg, SJL/J = 11.9 ± 1.4 mg, *n* = 14, *P* < 0.001; axillary node: CD-1 = 5.2 ± 0.7 mg, SJL/J = 8.3 ± 1.0 mg, *n* = 14, *P* = 0.016). The spleen also weighed more in the SJL: 179 ± 12.4 mg vs. 123 ± 8.7 mg, *P* < 0.01.

**Figure 3:**
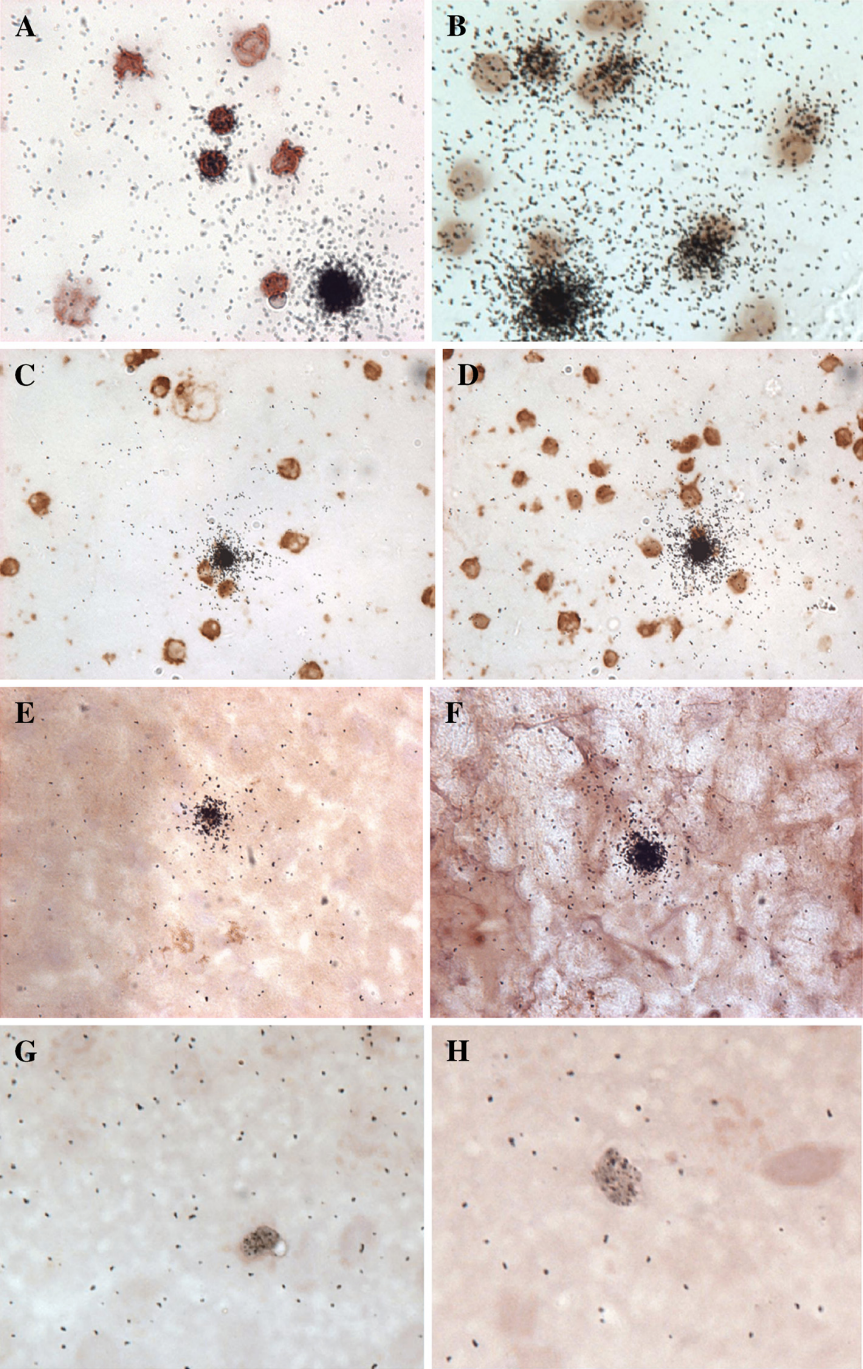
**Immunohistochemistry and autoradiography of **^**131**^**I-lymphocytes.** Cervical lymph node lymphocytes from SJL or CD-1 mice were radioactively labeled and injected intravenously into the autologous strain. After 30 min, cervical nodes or brain tissue were harvested, processed, and stained for CD45. Panel A shows ^131^I-lymphocyte preparation of cervical lymph node lymphocytes obtained from SJL female mice stained for CD45. Panel B shows stained ^131^I-lymphocytes prepared from the cervical lymph nodes of CD-1 males. Panels C and D show CD-1 cervical lymph node cells 30 min after the i.v. injection of ^131^I-lymphocytes. Panel E shows stained ^131^I-lymphocyte in cortex from SJL mouse. Panel F shows stained ^131^I-lymphocyte from hindbrain in SJL mouse. Panels G and H show CD45 stained ^131^I-lymphocytes from occipital cortex of CD-1 mice. All magnifications are at 40×.

To determine whether the radioactivity in our samples represented ^131^I-lymphocytes, we performed both autoradiography and immunohistochemistry on brain and lymph node tissue from CD-1 and SJL mice. Figure 3 shows the results of staining labeled cells with CD45 antibody. As a control and reference, panels A and B show purified ^131^I-lymphocytes prior to i.v. injection. Panels C and D show the cervical lymph node smear from CD-1 mice. Panels E and F show brain sections from SJL mice that received ^131^I-lymphocytes prepared from SJL mice. Panels G and H shows brain sections from CD-1 mice that received ^131^I-lymphocytes prepared from CD-1 mice. These results confirm that the radioactivity present in lymph node and brain tissue was attributable to ^131^I-lymphocytes.

**Figure 4:**
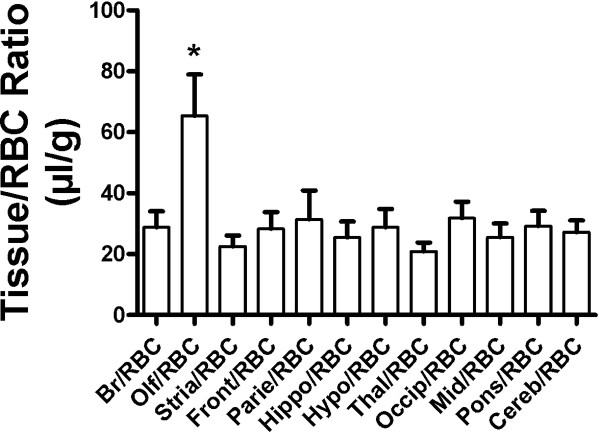
**Comparison among brain regions of uptake of **^**131**^**I-lymphocytes in CD-1 mice.** The olfactory bulb took up more ^131^I-lymphocytes than any other region. No other regions, including whole brain, were statistically different from one another. BR: whole brain; Olf: olfactory bulb; Stria: striatum; Front: frontal cortex; Parie: parietal cortex; Hippo: hippocampus; Hypo: hypothalamus; Thal: thalamus; Occip: occipital cortex; Mid: midbrain; Pons: pons-medulla: Cereb: cerebellum.

Figure 4 shows the distribution of ^131^I-lymphocytes among the brain regions in CD-1 male mice. ^131^I-lymphocytes entered all regions of the brain. A statistically significant difference was noted among the brain regions by ANOVA: *F*(22,71) = 3.11, *P* < 0.005. Newman-Keuls showed that the olfactory bulb differed from all other regions including the value for whole brain at *P* < 0.01. There were no other statistical differences among the regions.

**Figure 5:**
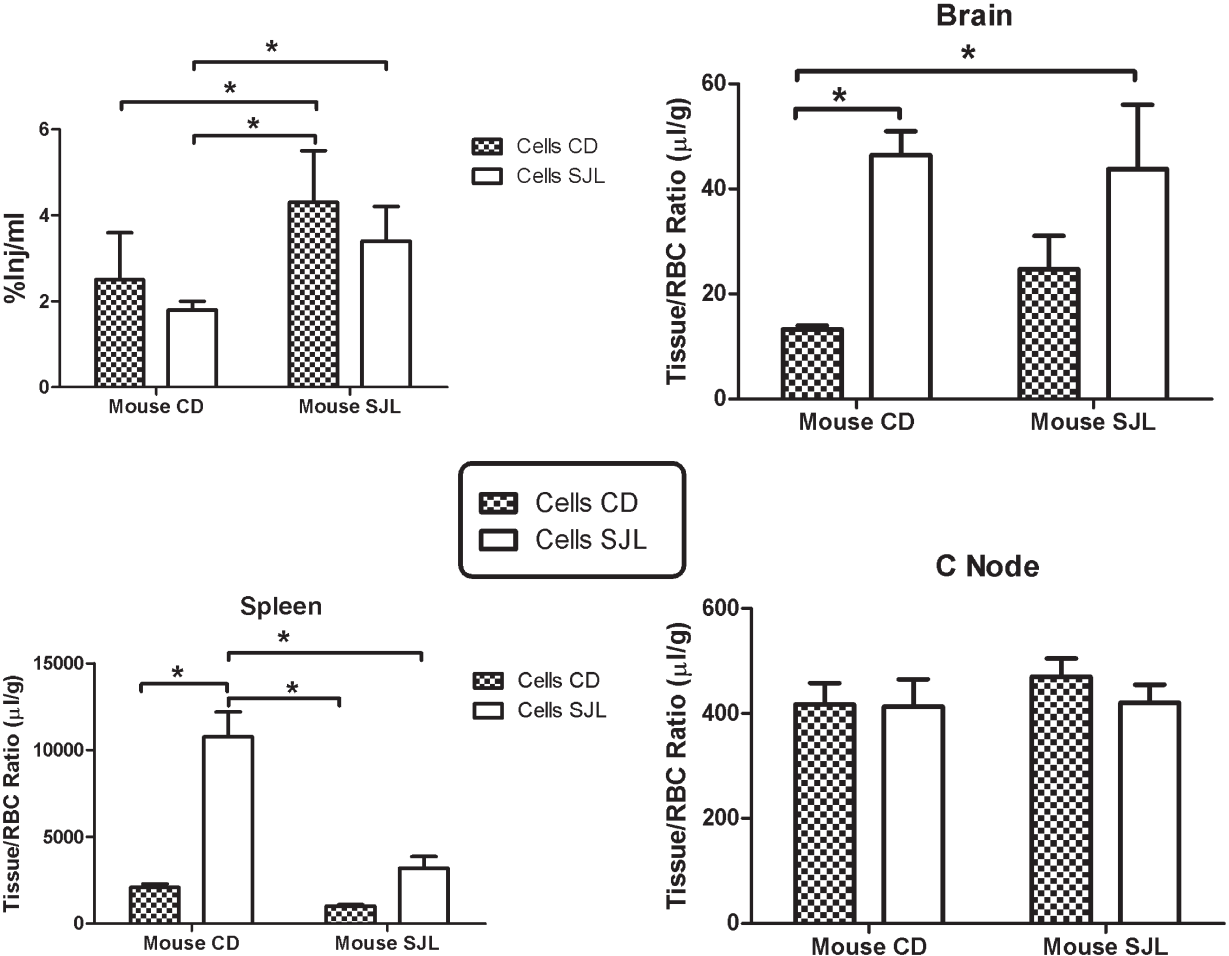
**Comparison of **^**131**^**I-lymphocyte tissue uptake in SJL female and CD-1 male mice.** A 2 × 2 design was used where lymphocytes from the cervical lymph nodes of SJL mice were injected into either CD-1 or SJL mice and lymphocytes from the cervical lymph nodes of CD-1 mice were injected into SJL or CD-1 mice. Upper right panel shows that SJL mice tended to have higher levels in the circulation of cells, regardless of source. Consistent with this, Cells (lymphocytes) accounted for only 10% of statistical variance, whereas the Mouse (tissues of uptake) accounted for 46%. Upper right panel shows higher uptake of Cells from either source into brain; statistical analysis showed that Cells accounted for 31% of variance. Lower left panel shows that Cells accounted for 40% of the statistical variance whereas Mouse accounted for 14%. Lower right panel shows uptake by cervical lymph nodes; there were not statistical differences among the four groups.

Figures 1 and 2 show that ^131^I-lymphocytes are taken up differently by the various tissues of the two strains. We wanted to know whether these differences were determined predominantly by the lymphocytes, by the tissues, or by interactions between the lymphocytes and tissues. We assessed the relative contributions of lymphocytes and tissues in determining these uptake patterns, using a 2 × 2 model. The 2 × 2 model was constructed by injecting ^131^I-lymphocytes from CD-1 mice into CD-1 or into SJL/J mice and by injecting ^131^I-lymphocytes from SJL/J mice into other CD-1 or SJL/J mice (*n* = 7/group). A two-way ANOVA was then performed on the four groups with Cells (lymphocytes) and Mouse (the strain receiving the i.v. injection) being the independent variables. Statistical significance (*P* < 0.05) was used to determine whether an independent variable had an effect on uptake and the degree of importance of the variable was indicated by the percentage of variance it explained. Thirty minutes after injection of the ^131^I-lymphocytes, the blood and tissues were collected. For blood, the two-way ANOVA conducted on %Inj/ml showed an effect of Cells (*F*(1,23) −5.27, *P* < 0.05) and Mouse (*F*(1,23) = 23.8, *P* < 0.0001), but not interaction (Figure 5, upper left panel). Mouse accounted for 46% of the variance whereas Cells accounted for 10%. These results show that although both lymphocytes and the tissues had statistically significant influences on ^131^I-lymphocyte clearance from blood, it was the tissues of uptake rather than the lymphocytes that exerted the predominant effect.

For brain, two-way ANOVA showed an effect of Cells (*F*(1,24) = 13.1, *P* < 0.005) but no effect of Mouse or interaction (Figure 5, upper right panel). Cells accounted for 34% of variance in brain uptake. These results show that it is the SJL lymphocyte and not the SJL brain that dictates the greater trafficking of the ^131^I-lymphocytes into the CNS.

For spleen (Figure 5, lower left panel), two-way ANOVA showed an effect for Cells (*F*(1,24) = 45.7, *P* < 0.0001), Mouse (*F*(1,24) = 29.2, *P* < 0.0001), and interaction (*F*(1,24) = 16.5, *P* < 0.001). Cells accounted for 40% of the variance, Tissue for 25%, and interaction for 14%. These results are most consistent with the lymphocytes having a major independent effect on determining uptake by spleen, but with the spleen and lymphocyte-spleen interactions also having effects.

Cervical and axillary nodes did not show any statistically significant differences attributable to cells or tissue (Figure 5, lower right panel; results shown only for cervical node). This is consistent with the results shown in Figure 2, in which the cervical and axillary nodes showed little or no differences in uptake of ^131^I-lymphocytes between the two strains. The results of the Newman-Keuls post-test are also shown in the Figure 5 panels (*P < 0.05).

**Figure 6:**
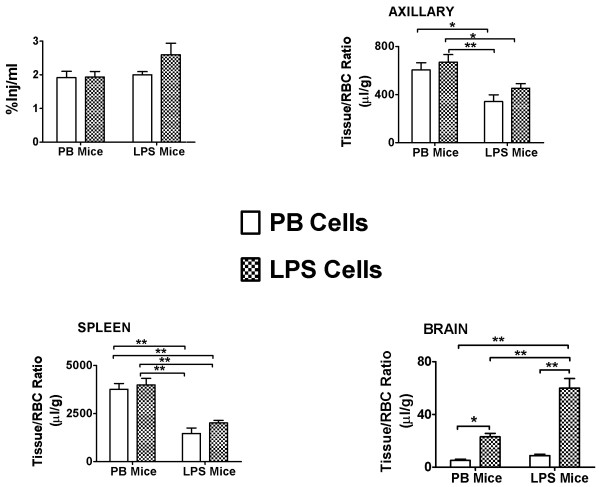
**Uptake of **^**131**^**I-lymphocytes in mice treated with LPS.** A 2 × 2 design was used, in which cervical-node lymphocytes from LPS-treated or phosphate buffer-treated (PB) mice were harvested, radioactively labeled, and injected i.v. into mice that were treated with LPS or into untreated mice. Upper left hand panel found no difference in levels of ^131^I-lymphocytes in the circulation. Upper right panel shows control mice took up more ^131^I-lymphocytes than did LPS-treated mice, with Mouse accounting for 42% of the statistical variance; these results show that it was the axillary node rather than the lymphocyte that dominated in determining the degree of uptake. Lower left panel shows similar results for spleen, with Mouse accounting for 72% of the variance. Lower right panel shows that LPS cells were taken up to a higher degree by brain. Statistical analysis showed that Cells accounted for 51% of variance, whereas Mouse accounted for 18% and interaction accounted for 12%.

A 2 × 2 study design was used to determine the effects of the endotoxin LPS on the uptake of lymphocytes in CD-1 mice (Figure 6). ^131^I-Lymphocytes from mice treated with PB or LPS (Cells) were injected into mice treated with PB or LPS (Mouse, *n* = 5 to 7/group). No statistical difference occurred in %Inj/ml (Figure 6, upper left panel) or for cervical nodes (data not shown). This suggests that LPS does not affect the net clearance of ^131^I-lymphocytes from the blood.

The LPS treatment reduced uptake of ^131^I-lymphocytes into the axillary node and spleen. For axillary nodes, there was a statistically significant effect only for Mouse: *F*(1,20) = 16.1, *P* < 0.001, which accounted for 42% of the variance, but no effect of Cells or interaction (Figure 6, upper right panel). Spleen also showed a significant effect only for Mouse: *F*(1,21) = 72.4, *P* < 0.0001, which accounted for 72% of the variance, with no effect of Cells or interaction (Figure 6, lower left panel). These results show that for axillary lymph node and splenic uptake, the effects of LPS on the tissue beds was the major factor in affecting ^131^I-lymphocyte uptake.

The LPS treatment greatly increased the uptake of ^131^I-lymphocytes into brain (Figure 6, lower right panel). The two-way ANOVA showed an effect of Cells (*F*(1,19) = 51.6, *P* < 0.0001), Mouse (*F*(1,19) = 17.9, *P* < 0.001), and interaction (*F*(1,19) = 12.1, *P* < 0.01); Cells accounted for 51% of the variance, Tissue for 18%, and interaction for 12%. The most consistent explanation for these results is that although brain, lymphocytes, and brain-lymphocyte interactions all play a measurable role in the LPS-induced increase in lymphocyte trafficking into brain, it is the lymphocyte that is the main determinant. The results for the Newman-Keuls post-test are shown in the Figure 6 panels (*P < 0.05; **P < 0.01).

## Discussion

These experiments applied a pharmacokinetic approach to examining the trafficking of immune cells across the BBB and into the CNS with comparison to uptake by spleen, cervical lymph nodes, and axillary lymph nodes. We validated the method by several techniques. These included examination by autoradiography with immunohistochemistry of the radioactivity taken up by the brain and cervical lymph nodes (Figure 3). To rule out the possibility that radioactivity in the brain or in the circulation had become detached from the lymphocytes, we used processing controls to show that the majority of radioactivity was deposited in the RBC and brain pellet and not in the supernatant (87% for the RBC pellet and 89% for brain), consistent with the radioactivity being attached to a cell. We also used capillary depletion to confirm that lymphocytes taken up by the CNS were recovered from the parenchyma and were not adhering to brain capillaries. Immune cells undergo a series of interactions with the brain endothelial cell in the process of entering the brain, including rolling or capture, arrest or firm adhesion, crawling, and diapedesis [10]. Loosely adhering lymphocytes would have been removed in the initial washout phase of this procedure and more tightly adhering lymphocytes and those engaged in diapedesis would have segregated in the capillary fraction. Under the conditions of the current experiment, about 80% of the ^131^I-lymphocytes detected in brain had crossed the BBB completely to enter the brain neuropile.

In CD-1 mice, we found that a small percentage of the immune cells crossed the BBB. The value averaged about 0.02%Inj/g, or about one cell in every 5,000 that was injected. Given that the typical CD-1 mouse has about 6,000 lymphocytes/μl of whole blood, a hematocrit of about 40% [21], and here a brain/RBC ratio of 10 μl/g for lymphocytes, we calculated a concentration of about 150,000 lymphocytes per g of brain or, given a brain weight of about 0.45 g, about 67,000 lymphocytes per whole mouse brain. Lymphocyte uptake by brain was readily evident at the first time point of 5 min, and rapidly reached a steady state. This quantifies the low level of trafficking across the BBB that many have postulated is occurring normally. This rate is too low to qualify immune cells as ‘flow dependent’; thus, this work suggests that immune cell trafficking into brain would be independent of the rate of cerebral blood flow [22-24].

Uptake was uniform throughout the brain with the exception of the olfactory bulb, which had an uptake about twice that of the other regions. This may reflect an increased immune surveillance of the olfactory bulb, the brain region most exposed to the outside environment owing to its projection as the olfactory nerve through the cribriform plate, a projection that molecules and pathogens can use as a pathway into the brain [25-27].

Trafficking into brain was higher in female SJL mice than in male CD-1 mice, regardless of whether results were expressed as %Inj/g-brain or as brain/RBC ratios. Immune cell trafficking can be more readily invoked in SJL female mice than in male SJL mice or other strains by the induction of experimental allergic encephalomyelitis (EAE), which is used as a model of multiple sclerosis and to study immune cell crossing of the BBB [28]. Therefore, we chose to compare the male CD-1, a commonly used strain of mouse for general studies, with the female SJL, the strain specially used for study of EAE and immune cell trafficking into brain, even though it introduced sex as a confounder. We found that naïve or untreated SJL female mice had a trafficking rate about 2 to 3 times higher than CD-1 males. The factors that predispose the SJL strains to the development of EAE are not altogether clear. Our current findings show that the SJL mouse has a predisposition to increased trafficking of immune cells into brain even in the absence of induction of EAE. This suggests that the induction process may work by accentuating processes that are already ongoing in the SJL mouse.

SJL and CD-1 mice also differed in the clearance rate of immune cells from blood. The CD-1 mice had a measurable clearance of ^131^I-lymphocytes from blood with a half-life of 171 min. In comparison, the SJL mouse did not show net clearance ^131^I-lymphocytes from blood. One explanation for these differences is that the exchange of ^131^I-lymphocytes between blood and tissues was at a steady state in the SJL mice, whereas with time an increasing number of ^131^I-lymphocytes entered the tissue beds in the CD-1 mice. For both strains, the calculated value at *t* = 0 was about log(0.5)%Inj/ml, which gives a Vd of about 32 ml. As the vascular space of these mice is about 2 ml, this shows that the majority of cells quickly distributed from blood into tissues. This shows that the vast majority of lymphocytes, about 97% of them, were in tissue beds rather than circulating. It might be presumed that much of this uptake was by the spleen and the lymph nodes. Multiplying the tissue/RBC ratio by %Inj/ml and dividing by 1,000 yields %Inj/g; multiplying %Inj/g by tissue weight yields the percentage of the injected dose contained by that tissue. For example, peak values for CD-1 spleen ranged from 15 to 20%Inj/g of tissue, a value about 500 times greater than the uptake rate into brain (Figure 1, upper panel). Since the spleen weighed, on average, 123 mg in CD-1 mice, the spleen sequestered about 2 to 2.5% of the injected lymphocytes. This shows that although lymphocytes were clearly being sequestered by the spleen, lymphocytes must have been entering many other tissues as well.

Because expression of results as %Inj/g of tissue is a calculation that includes %Inj/ml, we chose to present subsequent graphic results as tissue/RBC ratios rather than as %Inj/g of tissue. This is because the differences that occurred with time between the two strains in %Inj/ml would automatically bias towards differences in %Inj/g-tissue.

The spleen/RBC ratio for CD-1 mice was over twice the value of that for SJL. Thus, whereas the brains of SJL mice took up more ^131^I-lymphocytes than the brains of CD-1 mice, their spleens took up less. Minimal statistical differences were found for the tissue/RBC ratios between the strains for uptake by cervical or axillary lymph nodes. This shows that these two strains of mice differ in how their lymphocytes traffic into various tissues. It is also notable that although the lymphocytes were derived from cervical lymph nodes, their propensity for uptake by this tissue was no greater than and even less than for the spleen and axillary lymph nodes.

The differences in ^131^I-lymphocyte uptake between strains and among tissues raised the question of whether these differences were dictated by the lymphocytes or by the tissues that took them up. It is clear that immune cell trafficking into peripheral and CNS tissue beds requires an interactive cross talk between the endothelium and the immune cells [10,14,29]. The responsiveness of the brain endothelium is also influenced by cells behind the BBB, such as microglia and astrocytes [30]. However, whether cells or tissue beds could dominate under various conditions is a question that is difficult to address without methods that are temporal and quantitative. We investigated this question with a 2 × 2 experiment, in which cells from SJL or CD-1 mice were injected into either strain. We used measures of variance calculated by the two-way ANOVA to determine the relative influence of lymphocyte, tissue, and lymphocyte-tissue interactions in determining uptake. Figure 5 (upper left panel) shows that the %Inj/ml tended to be higher in SJL mice regardless of lymphocyte source. Statistical analysis confirmed this; although the differences in clearance from blood were found to be attributable to both Mouse (in this case, a proxy for clearance of ^131^I-lymphocytes from the circulation) and Cells (lymphocytes), Mouse accounted for 46% of the variance whereas Cells accounted for 10%. This argues that it is the peripheral tissues of uptake and not the lymphocytes that underlie the difference between SJL and CD-1 mice in clearance rate.

In contrast, lymphocytes from SJL mice entered the CD-1 and the SJL brain more rapidly than lymphocytes from CD-1 mice. The statistics confirmed this with only Cells showing a statistically significant effect; this demonstrates that it is the lymphocyte, not the brain and BBB, that primarily dictates the degree of trafficking into the CNS. For spleen, Cells accounted for 40% of variance, Mouse for 25%, and the interaction for 14%. Although SJL lymphocytes were taken up better by the spleen in both the CD-1 and the SJL mouse, the SJL mouse spleen took up lymphocytes regardless of source less readily than the spleen of the CD-1 mouse. In this case, then, it seems that while the SJL lymphocyte is more avid in its entry into splenic tissue than the CD-1 lymphocyte, the SJL spleen is less avid than the CD-1 spleen in its uptake of lymphocytes. Thus, both lymphocytes and the target tissues contributed to determining the degree of trafficking, but which of these dominated varied from case to case. Specifically, it was the peripheral tissues that were most important in determining clearance rate from blood, it was the lymphocytes that were dominant in determining the degree of uptake into brain and spleen, and there were no strain effects for uptake by cervical or axillary lymph nodes.

To test this observation further, that lymphocytes and tissues interact variably to dictate trafficking, we examined the effect of LPS in this 2 × 2 modeling design. LPS did not influence results for the %Inj/ml, suggesting that LPS does not induce altered clearance of ^131^I-lymphocytes from blood, at least not at the assessed 30 min time point. LPS did not affect uptake of ^131^I-lymphocytes by the cervical node, but LPS had statistically significant effects on uptake by the axillary node, spleen, and brain. Thus, the effect of LPS on lymphocyte uptake was tissue specific. In general, the spleen and axillary nodes of LPS-treated mice took up fewer lymphocytes (Figure 6, upper right panel and lower left panel) than those of non-LPS-treated mice. Consistent with this, the statistical evaluation for axillary lymph nodes and spleen found an effect for Mouse but not for Cells. In contrast, lymphocytes from LPS-treated mice had a higher uptake into brain than lymphocytes from non-LPS-treated mice. Statistical analysis found that Cells accounted for 51% of variance for brain uptake, while Mouse accounted for 18% and interaction for 12%. Thus, LPS caused a redistribution of lymphocyte preference by increasing brain uptake while decreasing splenic and axillary lymph node uptake. LPS thus affected uptake differently at the various tissues, increasing brain uptake by acting primarily on the lymphocyte, decreasing splenic and axillary lymph node uptake by acting primarily at those peripheral tissues, but having no affect on cervical lymph node uptake.

## Conclusions

In conclusion, we used pharmacokinetic methods and modeling based on the variance calculations of ANOVA to quantify trafficking of immune cells obtained from cervical lymph nodes into brain, spleen, axillary lymph nodes, and cervical lymph nodes. We found that the immune cells entered at a low rate into brain and that such entry occurred to a higher degree in the naïve SJL female than CD-1 male mice. Treatment with LPS also increased uptake into brain. Uptake by the immune tissues varied between SJL and CD-1 mice and between LPS-treated and untreated mice. Both lymphocytes and tissues influenced the degree to which lymphocytes were taken up by tissues, but one or the other tended to dominant in determining the degree of uptake. Lymphocyte characteristics chiefly accounted for increased immune cell entry into brains of SJL and LPS-treated mice and the decreased uptake into the spleen of SJL mice, whereas tissue had the primary influence in the decreased immune cell uptake by spleen and axillary nodes in LPS-treated mice.

## Abbreviations

ANOVA: analysis of variance; BBB: blood–brain barrier; CNS: central nervous system; EAE: experimental allergic encephalomyelitis; ^131^I-lymphocytes: mixed lymphocytes labeled with radioactive iodine; i.p: intraperitoneal; i.v: intravenous; LPS: lipopolysaccharide; LR: lactated Ringer’s solution; LR-BSA: lactated Ringer’s solution with 1% bovine serum albumin; NS: normal saline; PB: phosphate buffer; PBS: phosphate buffered saline; RBC: red blood cell; Vd: volume of distribution; %Inj: percentage injection.

## Competing interests

The authors have no competing interests.

## Authors’ contributions

All authors interacted in experimental design; experiments were conducted by WAB, MAE, and MLN; all authors participated in writing the manuscript. Authors have read and approved the final version of this manuscript with the exception of SSZ, whose untimely death occurred before final submission of the manuscript.
